# Intranasal administration of butorphanol benefits old patients undergoing H-uvulopalatopharyngoplasty: a randomized trial

**DOI:** 10.1186/1471-2253-15-20

**Published:** 2015-02-02

**Authors:** Lin Yang, De-feng Sun, Yue Wu, Jun Han, Ruo-chuan Liu, Li-jie Wang

**Affiliations:** Neuroelectrophysiology Lab, First Affiliated Hospital of Dalian Medical University, Dalian, 116011 P.R.China; Department of Anesthesiology, First Affiliated Hospital of Dalian Medical University, Dalian, 116011 China

**Keywords:** Butorphanol, Intranasal administration, Preemptive analgesia, H-UPPP, Early postoperative cognitive dysfunction

## Abstract

**Background:**

To evaluate intranasal administration of butorphanol on postoperative pain and early postoperative cognitive dysfunction in old patients undergoing H-uvulopalatopharyngoplasty (H-UPPP).

**Methods:**

A total of 260 male patients (65 to 77 years old) with obstructive sleep apnea hypopnea syndrome and scheduled for H-UPPP were divided randomly to receive intranasal butorphanol, intravenous butorphanol, intranasal fentanyl, or intravenous saline (controls). The definition of preemptive analgesia is that the tested drugs are given before anesthesia induction. Visual analog scale (VAS) and Bruggrmann comfort scale (BCS) scores were recorded at postoperative 1, 6, 12, 18, 24, 36, and 48 h. Postoperative cognitive dysfunction (POCD) was evaluated by Mini-Mental State Examination (MMSE) scores assessed one day before, and 1, 3, and 7 days postsurgery.

**Results:**

Compared with control group, those given preemptive analgesia required significantly less sufentanil during surgery, had less pain at postoperative 6–12 h; those given butorphanol experienced less nausea and vomiting, less pain at postoperative 6–24 h, and less POCD. Compared with patients given fentanyl, those given butorphanol required significantly less postoperative fentanyl, had less pain at postoperative 18–24 h, less nausea and vomiting, and less POCD. Compared with patients given intravenous butorphanol, those who received butorphanol by nasal route required significantly less postoperative fentanyl, had less pain at 36 and 48 h, and less POCD.

**Conclusion:**

Intranasal administration of butorphanol is safe and effective, reducing postoperative usage of analgesics and the incidence of POCD in old patients undergoing H-UPPP.

**Trial registration:**

ChiCTR-TRC-14004121.

## Background

Uvulopalatopharyngoplasty (H-UPPP) has been used increasingly for the treatment of obstructive sleep apnea-hypopnea syndrome (OSAHS). However, postoperative throat pain can cause a strong stress response that impairs the hippocampus, white matter, basal ganglia and prefrontal cortex, ultimately leading to postoperative cognitive dysfunction (POCD) [1–4]. In particular, elderly patients are at high risk of POCD [5]. Therefore, appropriate analgesia is necessary to prevent the development of POCD in elderly patients treated with H-UPPP.

Morphine and other opioid analgesics have been given for postoperative pain, but their use is often accompanied by nausea, vomiting, and other adverse reactions. Butorphanol is a derivative of morphinan that induces analgesia by activating the k-opioid receptor. It reduces the incidence of nausea, vomiting, and other postoperative side effects by partially antagonizing the -opioid receptor. The pain relieving effect of butorphanol is five times greater than that of morphine, without obvious activity on receptors [6, 7]. Our previous study showed that intravenous administration of butorphanol had good analgesia effects on patients after H-UPPP [8]. Other studies have shown that intranasal administration of butorphanol took only 20 min to achieve peak effect, with high absolute bioavailability [9]. If analgesic drugs have high absolute bioavailability, we expect decreased consumption of analgesics and diminished adverse reaction. By observing the effects of butorphanol via intranasal and intravenous injection on both postoperative analgesia and POCD, and comparing them with fentanyl intranasal administration and normal saline intravenous injection, we expected decreased consumption of analgesics, diminished adverse reaction and low incidence of POCD in elderly patients with H-UPPP.

### Aim of the study

The primary outcome is that intranasal administration of butorphanol could lower the incidence of POCD in old patients undergoing H-UPPP; therefore, the primary endpoint is POCD assessment within one week after the operation. Secondary outcome is that intranasal administration of butorphanol could reduce postoperative usage of analgesics.

## Methods

### Subjects

**Figure 1:**
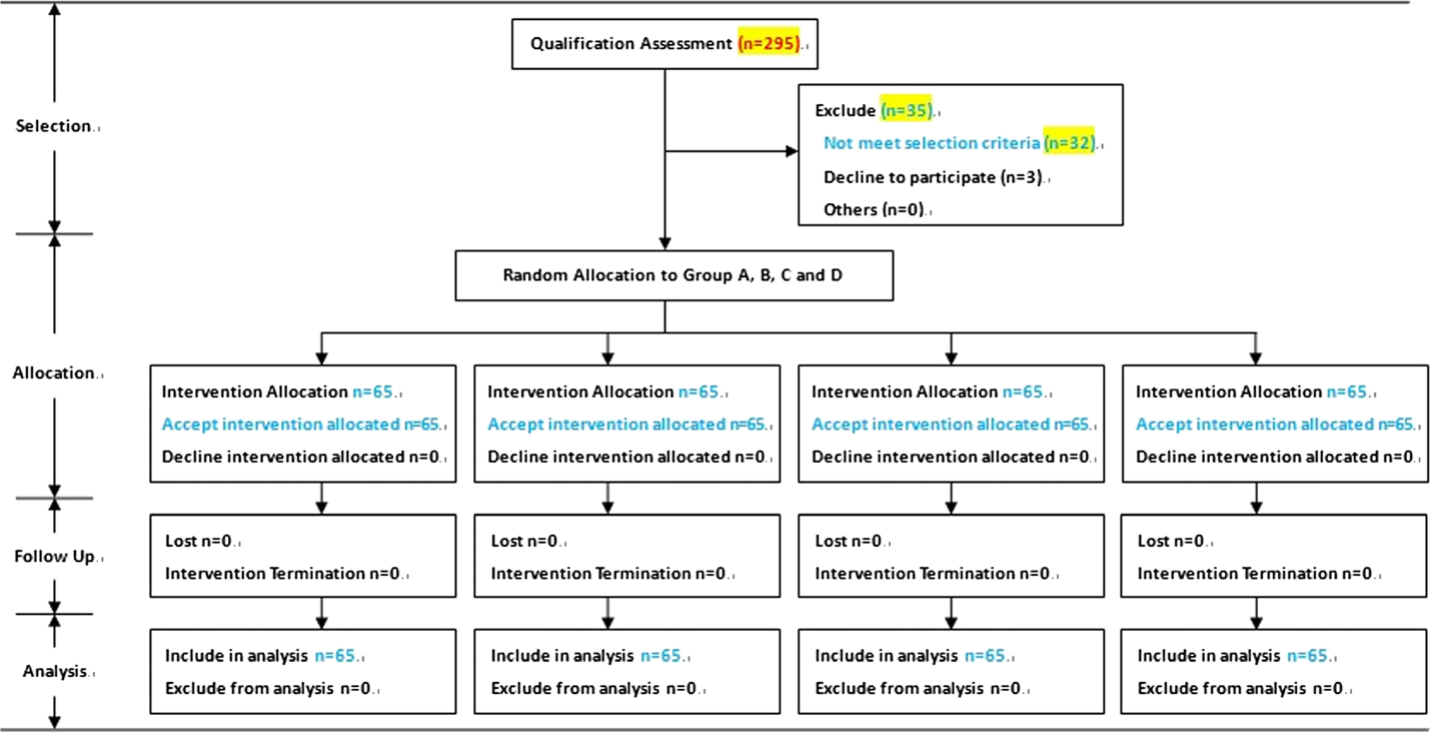
**The consort flow chart for RCT.**

The Ethics Committee of First Affiliated Hospital of Dalian Medical University approved this study. Patients or their families provided a signed informed consent form. This study was registered as ChiCTR-TRC-14004121. The consort flow chart for randomized controlled trial (RCT) was shown in Figure 1.

A total of 260 male patients (aged 65 to 77 y) with OSAHS who visited the first Affiliated Hospital of Dalian Medical University hospital between 1 January 2010 and 30 August 2014 were selected to undergo H-UPPP under general anesthesia. All patients weighed 88–111 kg, with BMI 28–35 kg/m^2^, apnea-hypopnea index 21–42 times/h, American Society of Anesthesiologists (ASA) physical status grade I or II, and the Mallampati airway classification grade I to IV based on the degree of exposure with pharyngeal in patients.

Rapid intubation was performed in patients with the Mallampati airway classification grades I or II, while awake intubation was performed in patients with the Mallampati airway classification grade III or IV. No patient had a history of drug use, and all had a simple mental state examination score > 23, junior high school or higher education, no significant history of neurological disease or hyperthyroidism, no liver or renal dysfunction, no severe visual or hearing impairment, and no recent use of sedatives, anti-coagulants, anti-depressants, or analgesics. Randomization was carried out with a computer-generated random number table. The allocations were concealed in consecutively numbered, sealed envelopes. Patients were randomly assigned into four groups (n = 65) based on the assignment found in the numbered envelopes as follows: intranasal administration of butorphanol (group A), intravenous administration of butorphanol (group B), intranasal administration of fentanyl (group C) and control group (group D).

Rapid intubation is the application of potent intravenous induction agent and quick acting muscle relaxants. It could enable patients to reach the unconsciousness and muscle paralysis in a short time to complete a tracheal intubation. Awake intubation refers to the tracheal intubation in patients with consciousness. The detailed method of awake intubation was as following:

-  Interpret to the patient properly, emphasizing cooperative matters, such as relaxing muscles, keep breathing, not to hold breath and not to feel nausea, in order to help the patient cooperate fully.
-  Apply 1% dicaine on throat tracheal surface anesthesia for 1–2 minutes.
-  To calm the patient, diminish throat reflection, intravenous inject Innovar (haloperidol 5 mg fentanyl 0.1 mg) by 1 ~ 2 ml for inducing.
-  The technique should be as light as possible, carry out the endotracheal intubation slowly and accurately.

### Sample size

Sample size calculation was performed based on the primary endpoint of POCD incidence. We assume that there is a 26% POCD incidence on control group and 3% on intranasal administration of butorphanol group. The selected sample size was adjusted for multiple comparisons using the technique of Bonferroni for controlling the overall type I error rate at 5%. The adjusted sample size of 63 patients per arm (totally 252 patients, including 10% drop-out rate) will have a power of 80% to detect difference between treatment arms, with a 2-sided alpha = 0.5.

### Anesthesia procedures

The patients had routine preoperative fasting for 8 h, and light liquid for 2 h, without preoperative treatment. Routine monitoring of bispectral index (BIS), blood pressure, heart rate, blood oxygen saturation, and invasive arterial pressure was performed during surgery.

Bispectral index was performed using an HXD-1 multifunctional monitor. Ten minutes before the induction of anesthesia, Group A received 2 mg nasal drip butorphanol (Novo Yang, Jiangsu Hengrui Medicine), Group B received 2 mg intravenous injection of butorphanol, group C received 0.1 mg nasal drip of fentanyl 0.1 mg (Yichang Humanwell Pharmaceutical), and group D received 2 mL intravenous injection of saline.

For Mallampati I and II patients in four groups, anesthesia was induced by intravenous injection of 0.05 mg/kg midazolam, 0.2 μg/kg sufentanil, 1 mg/kg propofol, and 0.3 mg/kg cisatracurium. For Mallampati III and IV patients in four groups, anesthesia was induced by awake fiberoptic intubation with intravenous injection of Innovar (haloperidol 5 mg fentanyl 0.1 mg) by 1–2 ml. After endotracheal intubation through the nose, mechanical ventilation was performed at a respiratory rate of 8–12 times/min, inspiratory to expiratory ratio 1:2, tidal volume 8–10 mL/kg, ventilation 100–120 mL/kg/min, and oxygen concentration 60-70%. During the operation, the tidal volume was adjusted to maintain an end-tidal carbon dioxide partial pressure of 35 mmHg (i.e., 4.65 kPa). For all patients in four groups, anesthesia was maintained by continuously intravenous injection of 0.2 μg/kg/h sufentanil, 0.15-0.30 mg/kg/h cisatracurium, and 2–3 mg/kg/h propofol.

Blood pressure was maintained fluctuations in mean arterial pressure to no more than 20% of base value by continuously intravenous injection of nicardipine and esmolol. The bispectral index was maintained between 50 and 60 by adjusting infusion speed of sufentanil and propofol. Within 15 min of the end of surgery the infusion of propofol was ended. The infusion of nicardipine and esmolol was ended after extubation.

Immediately after surgery, patient-controlled intravenous analgesia (PCIA) was administered using fentanyl, and the patient was moved into post-anesthesia care unit (PACU). Patients were extubated in PACU when the bispectral index was more than 90, patients were fully awake, muscle strength and oxygen saturation were the same as before surgery, and secretions and blood were cleaned from the mouth and throat.

### Intranasal administration

Intranasal administration was performed as described previously [10]. Briefly, the butorphanol dilution was dropped slowly into the side of the nose, to avoid going directly into the throat. The nasal cavity was filled with the butorphanol dilution and the patient remained in the original position for 1 min to fully absorb the butorphanol.

### Steward’s postanesthetic recovery scoring

Steward’s postanesthetic recovery scoring was performed as described previously [11]. Specifically, scoring was based on consciousness (awake, 2; responding to stimuli, 1; not responding, 0), airway (coughing on command or crying, 2; maintaining good airway, 1; airway requires maintenance 0), and movement (moving limbs purposefully, 2; non-purposeful movements, 1; not moving, 0).

### PCIA setting

Fentanyl as analgesics after surgery, a loading dose of 0.3 μ g / kg, background dose of 0.1 μ g.Kg^-1^.h^-1^, a single dose of 0.1 μg/kg, lockout time 15 min, use time of 48 h.

### Anesthesia monitoring

The operation time, PACU stay, and dosages of sufentanil, propofol, and fentanyl were recorded. Examinations were performed at postoperative 1, 6, 12, 18, 24, 36 and 48 h (T1-7), and the occurrences of postoperative pain, nausea, vomiting, pruritus, respiratory depression, dizziness, urinary retention, and other adverse reactions were documented. The mini-mental state examination (MMSE) scores were determined by psychiatrists one day before, and one day, 3 days and 7 days after the operation.

### Pain and comfort rating

Pain was rated using a visual analog scale (VAS) [12]. Comfort was rated using the Bruggrmann comfort scale (BCS) [13]. The pain after surgery was scored as: 0, persistent pain; 1, pain at rest; 2, no pain at rest but slight pain when swallowing; 3, no pain when swallowing; 4, no pain when the wounds were touched.

### POCD assessment

The MMSE score evaluation criteria consisted of: orientation, to time (5 points) and to place (5 points); memory, instant (3 points) and delayed (3 points); language ability, naming (2 points), narration (1 point), and writing (1 point); complex commands (5 points); and calculation (5 points); with a possible total of 30 points. The subjects were judged as having POCD when preoperative and postoperative MMSE scores differed by 2 points or more [14].

### Statistical analysis

Data are expressed as mean ± standard deviation and were analyzed using SPSS 15.0 statistical software. In-group were compared with analysis of variance for repeated measurement design. The differences between groups were compared by analysis of a sheffe’s test, enumeration data were compared with the chi-squared test. *P* < 0.05 was considered statistically significant.

## Results

### General characteristics of the patients

We found no significant differences in general condition, operation time, or the amount of propofol used in the four groups (*P* > 0.05). Compared with group D, PACU stay was shorter and the dosage of sufentanil was lower in groups A, B, and C (*P* = 0.041). In addition, postoperative dosage of fentanyl was significantly lower in group A than in groups B, C, or D (*P* = 0.049, Table 1).

**Table 1:** **Demographics, clinical features, and analgesics administered to the patients in the four groups**

	A	B	C	D
Age (y)	74 ± 3	73 ± 4	72 ± 4	72 ± 5
Weight (kg)	100 ± 11	99 ± 10	101 ± 8	101 ± 10
ASA class (I/II)	35/30	34/31	36/29	35/30
Hypertension or coronary heart disease (%)	29	31	28	31
Pulmonary disease (%)	14	15	17	15
Operative time (min)	115 ± 15	116 ± 14	119 ± 13	120 ± 12
PACU stay (min)	30 ± 6^a^	32 ± 4^a^	32 ± 5^a^	51 ± 9
Sufentanil (μg)	64 ± 12^a^	65 ± 10^a^	66 ± 9^a^	98 ± 11
Propofol (mg)	1379 ± 108	1382 ± 105	1377 ± 107	1381 ± 108
Postoperative fentanyl (g)	651 ± 65^a,b,c^	775 ± 76^a,c^	911 ± 101^a^	1232 ± 104

^a^Compared with group D, *P* = 0.041; ^b^compared with group B, *P* = 0.049; ^c^compared with group C, *P* = 0.047.

### Postoperative pain

**Figure 2:**
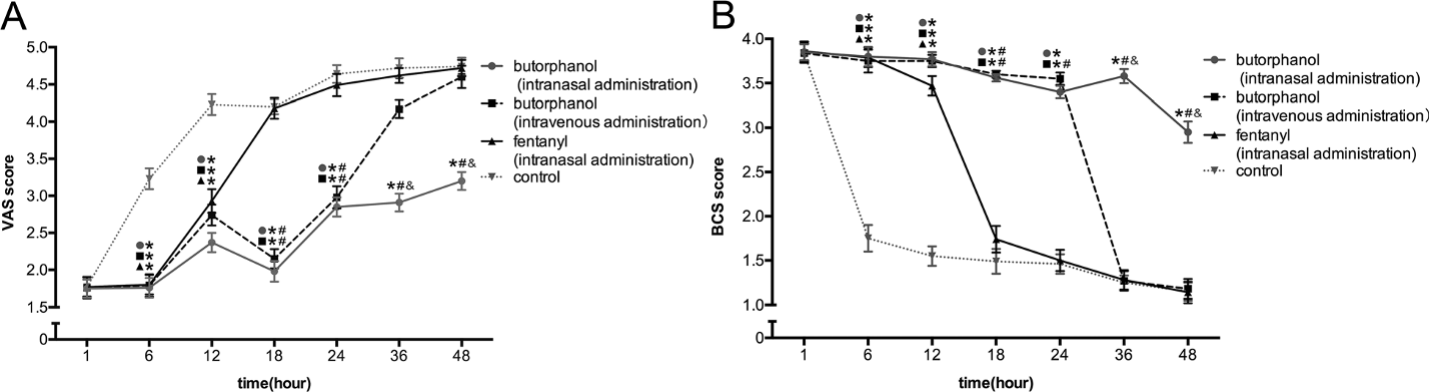
**Scores of VAS (A) and BCS (B) in four groups.** VAS scores were significantly decreased while BCS scores were significantly increased in group A at T2-7, group B at T2-5, and group C at T2-3, relative to the corresponding time points in group D (P = 0.041, each). Compared with group C at the corresponding time points, VAS scores were decreased while BCS scores were increased in group A at T4-7, and group B at T4-5 (P = 0.032). Compared with group B, VAS scores were decreased while BCS scores were increased in group A at T6-7 (P = 0.048).

VAS scores were significantly decreased while BCS scores were significantly increased in group A at T2-7, group B at T2-5, and group C at T2-3, relative to the corresponding time points in group D (*P* = 0.041, each). Compared with group C at the corresponding time points, VAS scores were decreased while BCS scores were increased in group A at T4-7, and group B at T4-5 (*P* = 0.032). Compared with group B, VAS scores were decreased while BCS scores were increased in group A at T6-7 (*P* = 0.048, Figure 2).

### Incidence of adverse reactions

Compared with group C and D, the incidence of nausea and vomiting was reduced in groups A and B (*P* = 0.031). However, the incidence of other adverse reactions showed no significant difference among the four groups (*P* > 0.05, Table 2).

**Table 2:** **Comparison of postoperative adverse reactions in the four groups (%)**

Group	Nausea, vomiting	Pruritus	Respiratory depression	Dizziness	Urinary retention
A	7.7^a,b^	0	0	12.3	0
B	9.2^a,b^	0	0	13.8	0
C	26.2	0	0	12.3	0
D	24.6	0	0	15.4	0

^a^Compared with group C, *P* = 0.029; ^b^compared with group D, *P* = 0.031.

### MMSE score and the incidence of POCD

Compared with T1, the MMSE score was significantly lower at T2 and T3 in groups B, C, and D (*P* = 0.041). Moreover, at T2 and T3, the MMSE score was significantly higher in group A and B than in group C and D, and MMSE scores were significantly higher in group A than in group B (*P* = 0.048, Table 3).

**Table 3:** **Comparison of MMSE scores in the four groups**

Group	T1	T2	T3	T4
A	28.2 ± 1.5	27.7 ± 1.8^a,b,c^	27.7 ± 1.9^a,b,c^	27.7 ± 1.9
B	28.0 ± 1.3	25.5 ± 1.9^b,c,d^	25.6 ± 2.0^b,c,d^	27.7 ± 2.0
C	27.9 ± 1.5	23.5 ± 1.7^d^	23.1 ± 2.0^d^	27.7 ± 1.9
D	28.1 ± 1.4	23.3 ± 1.7^d^	23.2 ± 1.8^d^	27.6 ± 2.0

^a^Compared with group B, *P* = 0.048; ^b^compared with group C, *P* = 0.042.

^c^compared with group D, *P* = 0.042; ^d^compared with T1, *P* = 0.041.

The incidence of POCD was 3.1%, 13.8%, 24.6%, and 26.2%, in groups A, B, C, and D, respectively. The incidence of POCD was significantly lower in groups A and B than in groups C and D, and it was significantly lower in group A than in group B (*P* = 0.043).

## Discussion

POCD indicates complications in the central nervous system and occurs frequently in elderly patients. It usually manifests as decline of memory, attention, language ability, and social interaction [15–17]. Age is one of the most significant factors influencing the incidence of POCD [5]. With increasing numbers of elderly patients who undergo surgery, POCD is an important cause of increased costs, prolonged hospitalization and recovery time, and decline in quality of life [4, 18].

Elderly patients often have hypertension, coronary heart disease, diabetes, lung disease, chronic bronchitis, and other age-related diseases and their metabolic function is poor. They thus have a significantly lower tolerance for surgery and anesthesia [19, 20]. Laalou et al. [21] reported that one week after surgery the incidence of POCD was 23% in patients aged 60 to 69 years and 29% in patients over 70 years. Newman et al. [22] reported that the incidence of POCD was 2 to 10 times higher in patients aged over 65 years than in younger patients, and three times higher in patients aged 75 years than in patients aged 65–75 years. It is important to identify the features of anesthesia that are associated with this side effect in elderly patients.

VAS and BCS are common methods used in clinical evaluation of the effects of postoperative analgesia, and they have good specificity and reliability [12, 13, 23, 24]. The MMSE method is commonly used in the evaluation of cognitive function, may exclude the emotional response to cognitive dysfunction, and is highly reliable and easy to conduct [14]. By using these methods in the present study we found that intranasal administration of butorphanol achieved good analgesia, effectively suppressed postoperative pain in elderly OSAHS patients who underwent H-UPPP, reduced postoperative analgesic dosage, and decreased the incidence of POCD. Intranasal administration of butorphanol was superior to intravenous delivery of butorphanol and intranasal administration of fentanyl.

Intranasal administration is a convenient, well-tolerated, non-invasive transmucosal route of administration. The nasal surface area is quite large, rich in submucosal vasculature, highly intertwined with arteries, veins and capillaries, and highly conducive to penetration and absorption of the drug. After administration through the nose, the drug can penetrate rapidly from the mucosa to blood circulation, avoiding gastrointestinal metabolism, hepatic degradation, and metabolism. In contrast, intravenous administration does not have these advantages and often causes nausea, vomiting, and other complications [25].

The better effects of intranasal administration of butorphanol compared with that of intravenous administration may be due to its higher absolute bioavailability. In the present study, the incidences of nausea and vomiting were significantly lower in groups A and B than in groups C and D. This may be related to the antagonism of butorphanol to the-opiod receptor. At T2 and T3, the mean MMSE scores were significantly higher in groups A and B than in groups C and D, and significantly higher in group A than in group B. The incidence of POCD was significantly lower in groups A and B than in groups C and D, and was significantly lower in group A than in group B. These data indicate that both routes of administration of butorphanol could reduce the incidence of POCD, but intranasal administration of butorphanol is superior to either intravenous administration of butorphanol or intranasal administration of fentanyl. This may be mainly due to the inhibition of the postoperative stress response, thereby reducing the incidence of aseptic inflammation in the brain which leading to the hippocampus, anterior cerebral white matter,basal ganglia and prefrontal cortex maintaining relatively good physiological function because of complete absorption after intranasal administration which produced high quality and duration of postoperative analgesia, and another reason for the lower incidence of POCD in group A is that the dosage of postoperative analgesics was significantly lower in group A than in the other three groups. In group C, despite intranasal administration fentanyl was metabolized faster than butorphanol. Thus the quality of analgesia was significantly worse than in group A, leading to higher incidence of POCD than in group A.

The limitations of this study are as follows: we focused on old and obese patients. In addition, butorphanol is used in an ORL minor procedure.

## Conclusions

In summary, for elderly patients with OSAHS undergoing H-UPPP, preemptive intranasal administration of butorphanol effectively suppressed postoperative pain, reduced the dosage of analgesics, and decreased the incidence of POCD. Intranasal administration of butorphanol was superior to vein administration of butorphanol or intranasal administration of fentanyl, and has good promise for clinical application.
